# Relationship Between Local Anesthetic Infiltration Pain Before Spinal Anesthesia and the Analgesia Nociception Index for Women Undergoing Elective Cesarean Delivery: A Prospective Observational Study

**DOI:** 10.7759/cureus.88671

**Published:** 2025-07-24

**Authors:** Hiroaki Kondo, Shunsuke Hyuga, Miho Shishii, Tomoe Fujita, Toshiyuki Okutomi

**Affiliations:** 1 Department of Anesthesiology, Kitasato University School of Medicine, Sagamihara, JPN; 2 Division of Obstetric Anaesthesia, Center for Perinatal Care, Child Health and Development, Kitasato University Hospital, Sagamihara, JPN

**Keywords:** analgesia nociception index, cesarean delivery, high-frequency vitality index, local anesthetic injection, postoperative pain

## Abstract

Background

Heart rate variability-derived indices, such as the high-frequency vitality index (HFVI)/analgesia nociception index (ANI), have been proposed as objective pain assessment tools. However, their ability to predict pain perception during local anesthetic injection (LAI) remains unclear.

Methods

This single-center observational study included 44 pregnant women scheduled for cesarean delivery under spinal anesthesia. The HFVI/ANI was measured for five minutes before LAI using a Root® monitor (Masimo Corp., Irvine, CA). The primary outcome measure was the mean HFVI/ANI before LAI during spinal anesthesia. The secondary outcomes included the area under the receiver operating characteristic curve to determine the cut-off and predictive accuracy.

Results

There was no significant difference in the pre-LAI HFVI/ANI between the patients with visual numeric rating scale (VNRS) of ≤3 and those with VNRS of >3 (HFVI: 64.25 ± 11.8 vs. 62.64 ± 12.3, P = 0.66). ROC analysis revealed poor predictive ability (AUC = 0.54, 95% CI: 0.36-0.72). No significant correlation was found between HFVI/ANI and VNRS.

Conclusion

Pre-LAI HFVI/ANI values were not associated with pain perception during LAI during cesarean delivery. Further studies are needed to explore optimal indicators for HFVI/ANI-based pain assessment.

## Introduction

The analgesia nociception index (ANI), which is based on heart rate variability, has been used as an objective indicator for evaluating acute pain after surgery in recent years [1]. This index is not limited to surgery under general anesthesia but is also used for evaluating pain during labor [2]. A high-frequency variability index (HFVI), which uses the same algorithm as ANI, was released in Japan in 2022 and can be displayed on the Root® monitor along with other physiological parameters [3]. HFVI/ANI analyzes heart rate variability caused by respiration, which is mediated by changes in parasympathetic nervous system stimulation to the sinus node of the heart. Pain stimuli cause a relative decrease in parasympathetic nervous system tone, resulting in a decrease in the HFVI/ANI score. HFVI/ANI scores are expressed on a scale of 0-100, with 100 indicating maximum parasympathetic nervous system tone and low nociceptive levels and 0 indicating minimum parasympathetic nervous system tone and high nociceptive levels [4]. HFVI/ANI is a highly useful point-of-care tool for perioperative pain management.

In obstetric anesthesia, pain severity during local anesthesia injection (LAI) before spinal anesthesia (not the pain caused by the needle piercing the skin) for cesarean delivery has been related to postoperative acute pain [5]. This correlates not only with the degree of postoperative acute pain but also with that of chronic pain [6]. It is known that pregnant women who strongly complain of LAI pain are at high risk of developing severe acute pain and chronic pain, but the specific population of pregnant women who will experience severe postoperative pain remains unclear. Given the clinical importance of identifying patients at risk, developing an objective, non-invasive method to predict LAI pain would be valuable. Furthermore, current pain assessments during LAI rely solely on subjective measures such as the numerical rating scale (NRS) reported by the parturient, which can be influenced by individual pain coping styles, emotional state, or communication barriers during labor. An objective, continuous physiological indicator such as HFVI/ANI could provide real-time, unbiased information about patients’ nociceptive response, potentially improving perioperative pain management and individualized analgesic strategies. One potential approach is the use of autonomic nervous system monitoring, as individual variations in baseline autonomic tone have been reported to influence pain sensitivity [7]. Specifically, individuals with lower baseline parasympathetic activity tend to exhibit greater sensitivity to noxious stimuli. Therefore, HFVI/ANI, which reflects parasympathetic activity, may serve as a potential predictor of pain. However, to date, no study has specifically examined whether these indices could predict the severity of LAI pain in pregnant women undergoing cesarean delivery.

We hypothesized that lower pre-LAI HFVI/ANI values, reflecting suppressed parasympathetic tone, would correlate with higher LAI pain scores. Therefore, in the present study, we aimed to investigate the correlation between HFVI/ANI and LAI pain in pregnant women undergoing elective cesarean delivery. Additionally, we sought to determine whether HFVI/ANI could predict which patients would experience moderate or severe LAI pain.

This research was previously presented at the 43rd Perinatal Symposium of the Japan Society of Perinatal and Neonatal Medicine, held in Tokyo from January 17 to 18, 2025.

## Materials and methods

Study design

This single-center, observational study was approved by the Kitasato University Medical Ethics Organization (B21-079) on June 29, 2021, and was conducted at the Kitasato University Hospital, Kanagawa, Japan, from June 2021 to December 2024. This trial was registered prior to patient enrollment in the University Hospital Medical Information Network Clinical Trials Registry (registration number: UMIN000045935, https://center6.umin.ac.jp/cgi-open-bin/ctr/ctr_view.cgi?recptno=R000052435). It was conducted and reported according to the Strengthening the Reporting of Observational Studies in Epidemiology (STROBE) statement [8]. Patients provided written informed consent for participation in this study before the day of cesarean delivery. The study was conducted in accordance with the principles of the Declaration of Helsinki.

Participants

The study included patients who underwent cesarean delivery; were nulliparous or primiparous; had an American Society of Anesthesiologists physical status score of 2; and were aged ≥ 20 years. The exclusion criteria were patients with difficulty or inability to communicate; patients scheduled for cesarean delivery under general anesthesia; patients with allergy to local anesthetics; patients with some kind of pain (those who have pain above a certain visual numeric rating scale (VNRS) threshold of any origin); patients taking analgesics; patients who were taking medication at baseline; patients with mental disorders requiring treatment; patients with abnormal pre-operative electrocardiograms; hypertensive disorders in pregnancy; and patients with a body mass index above 35.

Study procedure

An electrocardiograph, a noninvasive blood pressure measurement device, and a pulse oximeter monitor were attached to the patient after entering the operating room. Left lateral uterine displacement was performed using a pillow commonly used in our hospital, after which the patient was placed in the supine position. A sensor for monitoring HFVI/ANI (HFVI V1 Plus sensor; Mdoloris Medical System, Loos, France) was attached to the right anterior chest and left subcostal region of the patient. The average HFVIs/ANIs calculated over 120 and 240 seconds (HFVIi/ANIi and HFVIm/ANIm, respectively) were displayed on the screen of a Root® monitor (ver. V2.1.4.6 INT; Masimo Corp., Irvine, CA) via the HFVI MOC-9 module (Mdoloris Medical Systems).

The temperature in the operating theater was set to 27 degrees, no music was played (i.e., a quiet environment), and unnecessary entry and exit by staff was restricted. The HFVI/ANI (HFVI) was measured for five minutes after a five-minute rest (as recommended by the manufacturer). The position of the patients was changed to the right lateral decubitus position immediately after the measurement was completed. Spinal anesthesia was performed in the lateral decubitus position at the L3-L4 or L4-L5 interspace. The anesthetist performed LAI using a standardized method after sterile preparation with chlorhexidine gluconate and draping. Each participant was told that the anesthesiologist would inject a local anesthetic into her back just before the LAI was performed. While using neutral wording, ‘‘I am going to give you a local anesthetic in your back,” to minimize the chances for either a placebo or nocebo effect [9]. The local anesthetic used for the skin during spinal anesthesia was 1% lidocaine, which was filled into a 10-mL syringe. Using a 27G needle, a total of 2.0 mL of local anesthetic was injected over 30 seconds. The injection was administered from the skin surface and then deep into the skin. The needle was immediately removed after the injection. LAI was performed by multiple obstetric anesthesia specialists with comparable experience. A standardized study protocol was followed to minimize inter-operator variability. The LAI pain was evaluated using the VNRS. The VNRS asked the patient to rate the intensity of their pain on a scale from 0 to 10; 0 represented no pain, and 10 represented the worst imaginable pain [10]. The VNRS for LAI pain was assessed by nurses, who asked participants to rate the pain they experienced specifically during the injection of local anesthesia on a scale of 0-10. Participants were informed in advance that they would be asked to evaluate the pain associated with the injection, excluding the pain caused by the needle piercing the skin. Spinal anesthetics were administered using 0.5% hyperbaric bupivacaine 2.4 mL plus 10 μg fentanyl and 100 μg morphine hydrochloride.

Outcome measures

The HFVI/ANI was recorded while the patient was in the operating room. HFVIi/ANIi (reflecting short-term changes: over a 120-second period) and HFVIm/ANIm (reflecting mid-term changes: over a 240-second period) before surgery were extracted from the Root® monitor every two seconds using dedicated software (Trace v3025; Masimo Corp.). We mainly analyzed HFVIi/ANIi; HFVIm/ANIm was recorded but not analyzed. Measurements were taken for 10 minutes before spinal anesthesia began, and the average HFVIi/ANIi after five minutes of measurement was calculated.

Statistical analysis

With reference to a previous study [11], the sample size was 44 cases, assuming an alpha error of 0.05, power of 0.80, and a mean difference in ANI of 11±13 between the NRS ≤ 3 and NRS > 3 groups. Assuming a 30% dropout rate, the study was conducted with a target of 70 cases. We defined a LAI pain score of > 3 on the VNRS as moderate-to-severe pain [1,12]. We used Student's t-test for normally distributed quantitative data and the Mann-Whitney U and chi-square tests for non-normally distributed quantitative data. The normality of the data was visually assessed. The primary outcome measure was the mean HFVIi/ANIi before LAI during spinal anesthesia. An independent t-test was used to analyze the primary outcome. For the secondary outcome, receiver operating characteristic (ROC) curve analysis was performed to evaluate the threshold of the HFVI/ANI, which discriminates the severity of LAI pain (VNRS ≤ 3, VNRS > 3) evaluated using the VNRS. To confirm the prediction and diagnostic capabilities of the ROC curve, the area under the curve (AUC) and its 95% confidence interval (CI) were also determined. The optimal cutoff value was determined using the Youden index, which maximizes the sum of sensitivity and specificity. In addition, the correlation between HFVIi/ANIi and LAI pain was analyzed using the Pearson correlation coefficient. Two-tailed p-values of < 0.05 denoted statistical significance. EZR was used to perform the statistical analyses (Saitama Medical Center, Jichi Medical University, Saitama, Japan) [13]; it is a graphical user interface for R (The R Foundation for Statistical Computing, Vienna, Austria) and R Commander.

## Results

**Figure 1:**
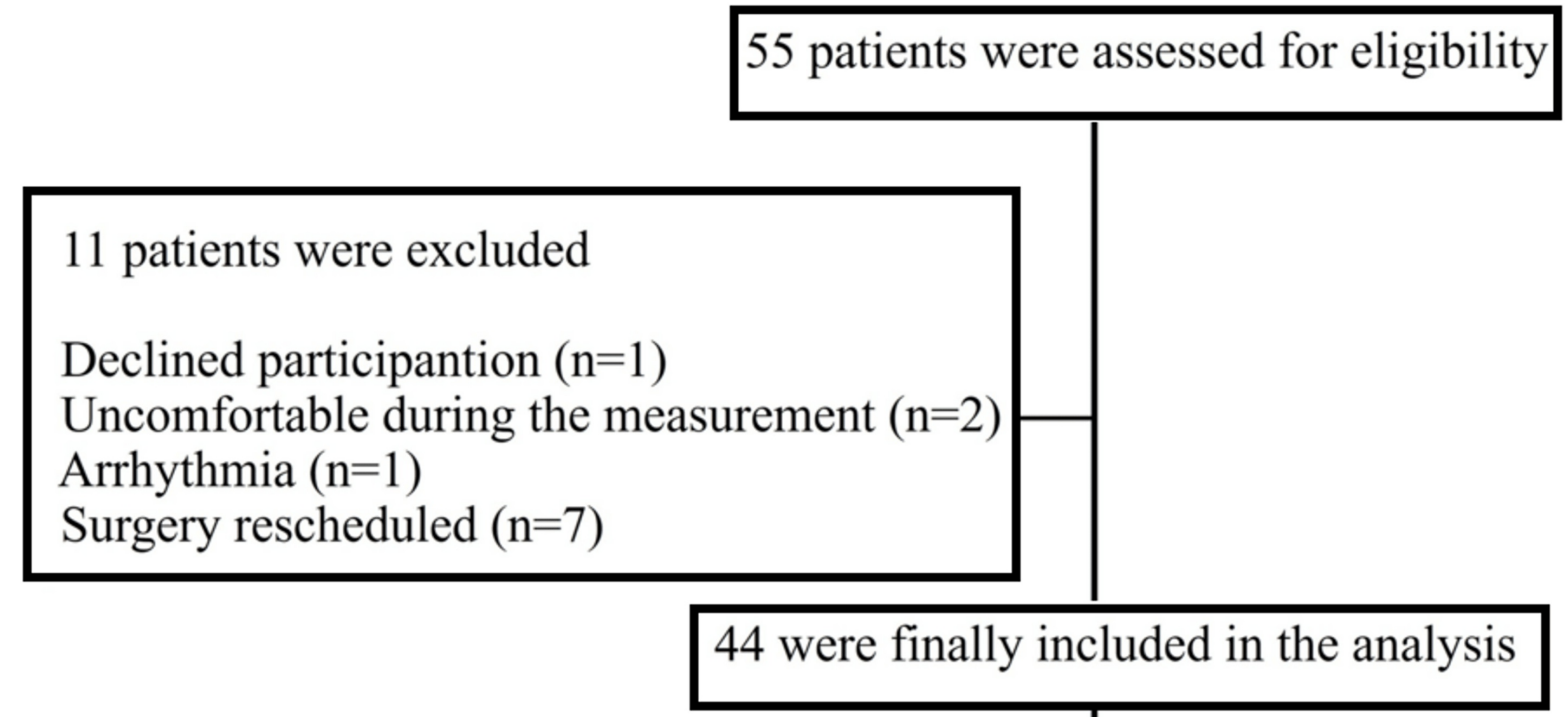
Participant selection The process of the selection for participants enrolled in the study.

Fifty-five patients met the inclusion criteria for this study. Eleven were excluded before HFVI/ANI data collection. As a result, the data from the remaining 44 patients were analyzed (Figure 1). The characteristics of the patients are presented in Table 1. There were no missing data on the HFVI/ANI just before the LAI was performed.

**Table 1:** Demographic and obstetric data Data are presented as mean ± standard deviation (SD) or median (interquartile range, IQR). BMI: body mass index; CD: cesarean delivery

Variables	N = 44
Maternal age (yr), mean (SD)	35.6 (5.2)
Body weight (kg), mean (SD)	63.2 (8.0)
		Height (cm), mean (SD)	158.6 (6.0)
Maternal BMI (kg/m^2^), mean (SD)	25.1 (2.7)		
		Gestational age (weeks), median (IQR)	38 (37–38)
Gravida (n, %)			
1	14 (31.8)
2	17 (38.6)
≥3	13 (29.5)
Previous CD (n, %)	
Yes	23 (52.2)
No	21 (47.7)

Primary outcome

**Figure 2:**
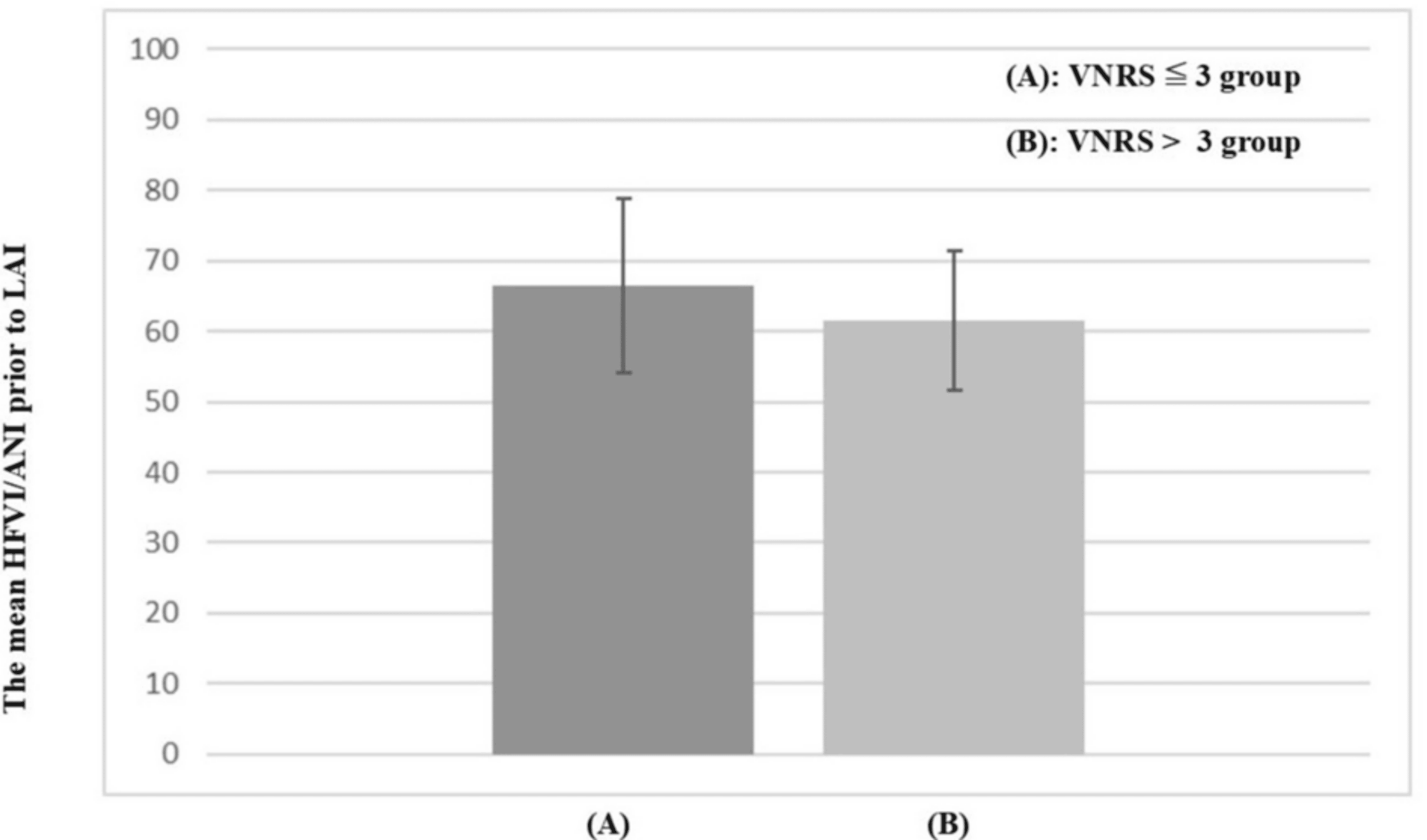
The mean HFVI/ANI before LAI in the two group The mean HFVI/ANI before LAI for the (A) VNRS ≤ 3 and (B) VNRS > 3 groups. Bar chart shows the mean HFVI/ANI + standard deviation (SD). No significant differences between the groups were detected. ANI: analgesia nociception index; HFVI: high-frequency variability index; LAI: local anesthesia injection; VNRS: visual numeric rating scale

The mean (standard deviation (SD)) HFVIi/ANIi before the LAI was 64.25 (11.8) and 62.64 (12.3) for the VNRS ≤ 3 and VNRS > 3 groups, respectively (difference in means: 1.61; 95% confidence interval (CI): -5.72 to 8.93; P = 0.66). There was no significant difference between the HFVIi/ANIi values just before the LAI was performed (Figure 2).

Secondary outcome

**Figure 3:**
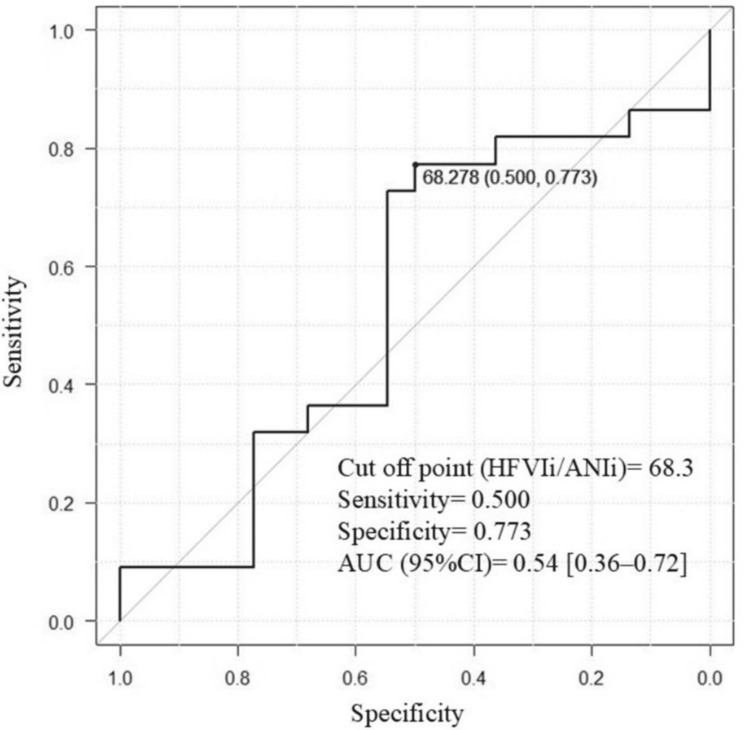
The predictive value of HFVI/ANI for LAI pain ROC curve analysis was used to obtain the optimal cut-off for HFVIn/ANIn before spinal anesthesia to predict LAI pain (NRS > 3). ANI: analgesia nociception index; AUC: area under the receiver operating characteristic curve; HFVI: high frequency variability index; NRS: numerical rating scale; ROC: receiver operating characteristic

The ROC curves used to obtain the optimal cut-off HFVIi/ANIi values before the LAI were used to predict VNRS > 3 and VNRS ≤ 3. The optimal cut-off point was 68.3, sensitivity was 0.773, specificity was 0.500, and the AUC (95% CI) was 0.54 (0.36-0.72) (Figure 3).

**Figure 4:**
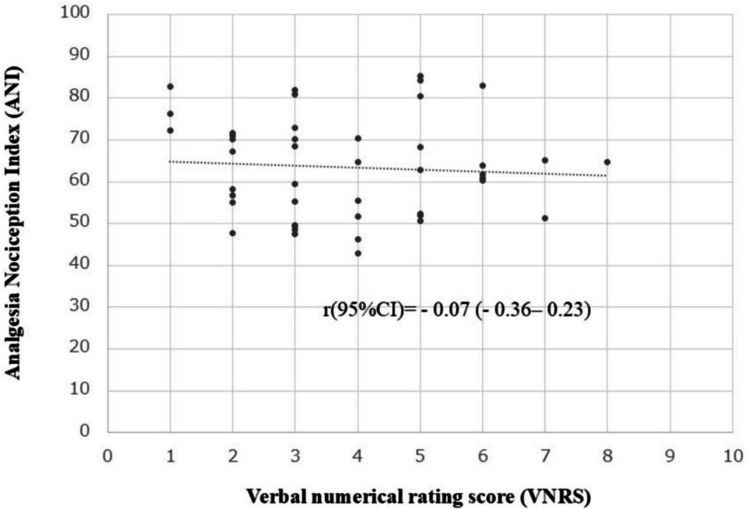
Scatter plot of VNRS and HFVIi/ANIi before the LAI was performed

Scatter plots showed no association between the VNRS and HFVIi/ANIi values before the LAI was performed (Figure 4).

## Discussion

The present study investigated whether HFVI/ANI is related to LAI pain. However, we found no statistically significant difference in the HFVI/ANI between pregnant women who reported moderate or severe pain (VNRS > 3) and those who reported mild pain (VNRS ≤ 3) during LAI for spinal anesthesia. Additionally, ROC curve analysis indicated that HFVI/ANI lacked sufficient predictive value, and the estimated regression model showed no correlation between HFVI/ANI and LAI pain.

ANI has been reported to be useful in obstetric populations. For example, ANI is related to pain scores during labor in conscious, spontaneously breathing pregnant women [2]. In addition, ANI is used not only in association with postoperative pain but also as a predictor of adverse events [14,15]. ANI has been shown to be useful for predicting maternal hypotension and fetal heart rate abnormalities when combined spinal and epidural analgesia is performed during labor [14]. Similarly, it has also been reported that measuring HRV before neuraxial anesthesia in pregnant women undergoing cesarean delivery can be used to predict hypotension after spinal anesthesia is performed [15].

Although previous studies have demonstrated that HFVI/ANI is associated with pain intensity during labor and hemodynamic changes in obstetric populations, there is limited evidence regarding its relationship with acute procedural pain, such as LAI pain. However, as HFVI/ANI reflects autonomic nervous system activity and has been shown to correlate with both pain intensity and nociceptive stimuli, we hypothesized that baseline HFVI/ANI values could be related to susceptibility to LAI pain in pregnant women undergoing cesarean delivery. In contrast to expectations, HFVI/ANI was not related to LAI pain and was not identified as a useful predictor of LAI pain severity in the present study.

In fact, this is in line with previous studies investigating whether HFVI/ANI can be used as a predictive indicator of postoperative pain. A previous study [16] assessing whether HFVI/ANI before extubation in patients under general anesthesia and peripheral nerve block could predict postoperative pain also found no significant association. Combined with our findings, this suggests that HFVI/ANI may not be a reliable predictor of pain intensity.

However, the potential utility of objective physiological indicators for pain assessment in obstetric anesthesia remains an important issue. As subjective pain scales, such as the NRS, can be influenced by individual pain thresholds, cultural and emotional factors, and communication barriers, objective measures like HFVI/ANI have the potential to serve as complementary tools that can detect or quantify pain responses in real time without relying on verbal feedback from patients.

Some limitations of this study might underlie why ANI could not be identified as a predictive indicator of LAI. First, in our study, HFVI/ANI was measured in a resting state before LAI. Notably, the average resting ANI in healthy volunteers was reported as 82.1±10.7 in a study [17], whereas the average ANI in our study population was approximately 60. HFVI/ANI can be influenced by various environmental stimuli beyond pain, including background noise, blood pressure monitoring, anxiety, and excitement. These factors might have influenced HFVI/ANI measurements of our conscious patients. Second, we measured HFVI/ANI in the obstetric operating room, with left uterine displacement positioning and concurrent vital sign monitoring, which may have served as confounding factors. Noise and anxiety were not controlled for, possibly reducing the reliability of HFVI/ANI as a pain indicator. Measuring baseline HFVI/ANI in a controlled environment, such as a quiet inpatient room, before cesarean delivery may provide more accurate evidence regarding its potential as a pain prediction tool. In addition, the needle size used for LAI in this study differed from those used in previous studies (27G [5] vs. 24.25G [6]), which might have influenced pain perception due to differences in injection pressure. Moreover, the sample size of this study may not be sufficient. While postoperative pain has been evaluated using HFVI/ANI [1,11,12], no studies have analyzed LAI pain using HFVI/ANI, making it difficult to estimate the appropriate sample size. Therefore, we applied the effect size obtained from HFVI/ANI for postoperative pain assessment to calculate the sample size for this study.

## Conclusions

In this study, there was no statistically significant difference in HFVI/ANI between the VNRS > 3 and VNRS ≤ 3 groups just before the LAI was performed. The HFVI/ANI derived from the ROC curve did not have sufficient predictive value, and the estimated regression model also did not show a correlation between HFVI/ANI and LAI pain. Further research is needed to determine whether HFVI/ANI can be used as an objective indicator to distinguish and quantify the intensity of pain in pregnant women without using pain scales such as the NRS.
